# Report on Obstetrics and Gynæcology

**Published:** 1874-04

**Authors:** A. Reeves Jackson

**Affiliations:** Lecturer on Gynæcology, Rush Medical College, Chicago


ARTICLE II.—*Report on Obstetrics and Gynæcology.* By A. REEVES JACKSON, M.D., Lecturer on Gynæcology, Rush Medical College, Chicago.

1. *Fortnightly Hemorrhage during Pregnancy.* By Dr. S. HAYNES. (Brit. Medical Journal, Nov. 29, 1873. American Journal of the Medical Sciences, Jan., 1874.)

2. *The Forceps in Midwifery.* By Dr. JAMES MOORE. (London Lancet, Jan., 1874.)

3. *The Influence of Malposition of the Uterus upon Sterility.* By Dr. HERMANN BEIGEL.* (Obstet. Journal of Great Britain and Ireland, Feb., 1874.)

1. That a sanguineous discharge, having a rhythmic character, may occur during pregnancy, is now admitted by all obstetricians. It is also certain that such cases are not so exceptional as was formerly supposed by most authors. In 1858, Dr. Elsasser published† a contribution upon this topic, founded upon fifty cases, extracted from the records of the Stuttgart Lying-in Hospital, and

* Ueben den Einfluss der Lagereranderugen der Gebarmutter auf die Sterilitat, von Dr. Hermann Beigel.

† Monatschrift fur Geburts—Kunde. Bd., 73.

said to rest upon the most accurate information. In the fifty cases the discharge occurred in the following manner; once in eight, twice in ten, three times in twelve, four in five, five in six, eight in five, and nine in two. The peculiarities of the rhythm were inquired into in thirteen of the cases. It occurred regularly every four weeks in four, every sixth week in one, and in the others it is not clearly stated. In one case it appeared first in the middle of gestation, and henceforth came on every four weeks, lasting each time three or four days.

The particulars of Dr. Haynes' case were read before the Worcester Medical Society. The patient had eight children, all carried to full time, and born alive, perfect. In all her pregnancies there had been a hemorrhagic uterine discharge, more profuse than her menses, (which were normal in quantity and quality), but of exactly the same nature, every fortnight up to the sixth month, whence, until labor, there was no loss. Each of these discharges was preceded by a few days very severe headache, and was accompanied by severe dorsal pain, and bearing-down sensations. She never had any leucorrhœa. When not pregnant her catamenia were regular, recurring monthly. An abundant bloody discharge, occurring every fortnight, was therefore her test of pregnancy.

Treatment, position and rest had no influence in lessening the amount of the discharge. She does not lose much after confinements, and she presents none of the ordinary indications of the hemorrhagic diathesis.

Dr. Elsasser, in commenting upon the cases reported by him, states that these discharges take place more frequently during the first half of pregnancy, and especially in the early months of this, than during the latter half.

The nature and source of these anomalous discharges have been the subjects of much variance of opinion. If the prevalent theory, which regards the menstrual flow as dependent upon, and an epiphenomenon of ovulation, be correct, then we must look upon these cases as pathological rather than physiological. If, however, as some still maintain, menstruation, or the menstrual flow, is nothing more than a periodical hemorrhage from the lining membrane of the uterine body, not caused by, or necessarily connected with the maturation and dehiscence of the ovule, then we may consider these cases as normal, although exceptional in their character.

During the early months of pregnancy, prior to the occlusion of the cavity of the body of the uterus by the contact and coalescence of the decidua vera and reflexa, there can be no difficulty in understanding that the discharge might proceed from its usual source, the lining membrane of that portion of the organ. This occlusion does not take place until the third month, and prior to that time, the open state of the fallopian tubes and of the cervix uteri present no more obstacles to the exit of the discharge than are present at each ordinary menstrual period.

Dr. H. R. Storer* and Koeberle† have each published a case, in which both ovaries and a large portion of the uterus were removed, yet, in that of the former there appeared, twenty-six days after the operation, a sanguineous effusion, attended by the usual accompanying sensations of menstruation, and in that of the latter "there was no interference with the menstrual flux." Therefore, if a small portion of the cervix uteri may be sufficient to furnish a regular persistent bloody discharge, we may find in the fact a solution of at least some of the cases in which such discharge has been said to continue throughout the entire period of utero-gestation.

2. Dr. Moore maintains that obedience to the rule of the older teachers, that the use of the forceps should not be had recourse to so long as the fœtus makes *any* advance, is fraught with evil both to the mother and child. He avers that the cases assisted by the employment of the instrument get about sooner and feel better than the unassisted ones do, and that by its "more frequent and more timely use we would not only lessen the maternal and infantile death rate, but by eliminating all or most of the causes of death and other pathological states, we would bring our patients nearer a healthy standard of recovery."

He concludes by enumerating the classes of cases in which he thinks it justifiable and obligatory to use the forceps, as follows:

(1.) In *all* cases where the first stage of labor is completed and the head stationary in a position favorable for their use. Under these conditions he would not wait longer than two hours.

* *American Journal of the Medical Sciences*, Jan., 1866, p. 110.

† Liegois on the "Function of Reproduction," Paris, 1869.

(2.) In cases where, though the head is advancing, the labor is tedious, from the fact of the pains being too weak or having almost ceased.

(3.) In cases where the pains are stronger than is warranted by the advance made.

(4.) In hemorrhage, if excessive; and in some cases of convulsions.

(5.) In cases, favorable for operation, where the patient is very desponding or impatient.

(6.) In cases of a tedious nature where there is a rigid fourchette or a lengthened perineum, especially where the pains are not steady in rhythm and force.

(7.) In cases of occipito-posterior positions, unless the labor is advancing quickly.

(8.) The second twin, if a head presentation, and not advancing quickly after the first.

(9.) To save time, if the case is favorable for forceps. In such cases, Dr. Moore does not scruple to use the means at his command, of relieving the woman from travail and himself from work.

3. Dr. Beigel bases his paper upon examinations, made by himself, of 125 sterile women. In 114 of these it was not difficult to assign the cause of the sterility; 34 had some form of malposition of the uterus; 26 had versions; 12, flexions; and 2, prolapsus. A deviation of the uterus from its normal position, whether flexion or version, does not, in itself, cause any impediment to conception; yet such a malposition may render it difficult, since it prevents the onward movements of the spermatozoa. This impediment is not, however, necessarily one which cannot be overcome; but it is otherwise, when, as is not seldom met with in flexion, the anterior lip of the os is longer than the posterior, and is so firmly fixed against the posterior wall as more or less to block up the passage, and thus form a barrier to the advance of any foreign body. Therefore, we must not so much consider the degree of the version as the determining cause of the sterility, as the relation of the lip of the os to the anterior or posterior wall of the vagina, according as we have to do with retro- or ante-version. Likewise, a pronounced bend in the uterus does not *per se* necessitate sterility, for the canal of the uterus may still be permeable; but sterility follows when the flexion is such that the two walls of the cervix, or of the uterus, are in contact, so that the canal is quite closed. It is in such cases as these that the sterility persists so long as the position of the uterus is such as to close the canal. The author therefore considers that the only rational treatment is to replace the uterus in its normal position. For this purpose he has used, with advantage, an intra-uterine pessary of the following form: It consists of an india-rubber ball, to which a perforated uterine stem is fixed. It is introduced by passing the sound through the tube and the india-rubber ball. After it is in position, the ball is blown up to its proper size, and the tube from it is fixed to a girdle.

